# Inflammatory Myofibroblastic Tumor of the Bladder in an Adolescent Girl: A Case Report on Successful Management With Partial Cystectomy

**DOI:** 10.7759/cureus.77095

**Published:** 2025-01-07

**Authors:** Annem Haritha, Sri Balram A, N Rama Murthy

**Affiliations:** 1 College of Medicine, Mamata Academy of Medical Sciences, Hyderabad, IND; 2 Department of Urology, Mamata Academy of Medical Sciences, Hyderabad, IND; 3 Department of Urology, Mamata Medical College, Khammam, IND

**Keywords:** adolescent, hematuria, histopathology, immunohistochemistry, inflammatory myofibroblastic tumor, partial cystectomy, transurethral resection of bladder tumor, urinary bladder

## Abstract

Inflammatory myofibroblastic tumors (IMTs) are rare bladder tumors that are difficult to differentiate from other malignant bladder tumors because of their non-specific presentation. Histopathology and immunohistochemistry serve as the main diagnostic tools in such cases. They have a very low malignant potential but can frequently recur. However, there are limited guidelines with regard to the choice of treatment for these tumors. This case scenario pertains to a 16-year-old female patient who had gross hematuria and fever, devoid of additional symptoms. Further evaluations uncovered a neoplastic mass lesion emerging from the left dome of the urinary bladder. Following transurethral resection of the bladder tumor, histopathological examination was consistent with an IMT. The patient underwent a partial cystectomy, and the diagnosis was established by histopathology and immunohistochemistry. Vigilant monitoring, post-surgery showed no signs of recurrence or metastasis during the six-month and one-year follow-up. This case underscores the significance of a precise diagnosis of the rare bladder tumor and emphasizes the need for tailored treatment to ensure comprehensive care and prevent recurrence.

## Introduction

The occurrence of an inflammatory myofibroblastic tumor (IMT) within the bladder is rare; however, its manifestation typically involves symptoms indicative of lower urinary tract dysfunction, such as hematuria and bladder outlet obstruction [1]. It is often locally aggressive, presenting in a similar way to urothelial carcinomas of the bladder [2]. It can occur in a wide range of age groups but is most often seen in children, adolescents, and young adults, with a slight predominance in women [3]. It is imperative to differentiate this tumor from alternative malignant spindle cell tumors, including the sarcomatoid variant of urothelial carcinoma, leiomyosarcoma, and benign lesions like postoperative spindle cell nodules of the bladder, as the treatment approach significantly diverges depending on the specific tumor type [3]. The exact etiology of IMTs remains elusive, with theories suggesting an inflammatory response to infection or an underlying, low-grade malignancy [3]. Despite its typically slow-growing nature, IMT poses a distinct diagnostic challenge due to its resemblance to aggressive malignant neoplasms, necessitating histopathological examination of specimens for conclusive identification.

## Case presentation

**Figure 1:**
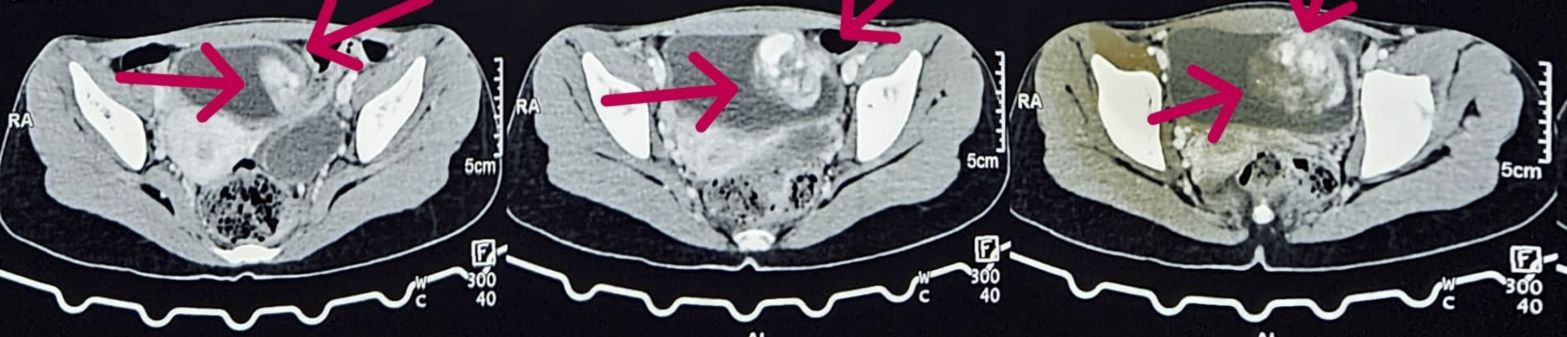
Contrast CT showing mass lesion arising from the left dome of the bladder.

A 16-year-old female patient presented with a history of hematuria for three months accompanied by fever for one week, devoid of other complaints, medically treated elsewhere earlier, with no improvement in symptoms, no significant family history, and no palpable masses or lymph nodes. Following a comprehensive series of diagnostic tests, including a complete blood picture, complete urine examination, renal function tests, liver function tests, and abdominal ultrasonography, the findings revealed the presence of blood and proteins in urine, severe anemia with a hemoglobin level of 6.1 mg/dl for which the patient had undergone multiple blood transfusions, while a CT urogram identified an intense, heterogeneously enhancing mass lesion originating from the left dome of the urinary bladder (Figure 1).

**Figure 2:**
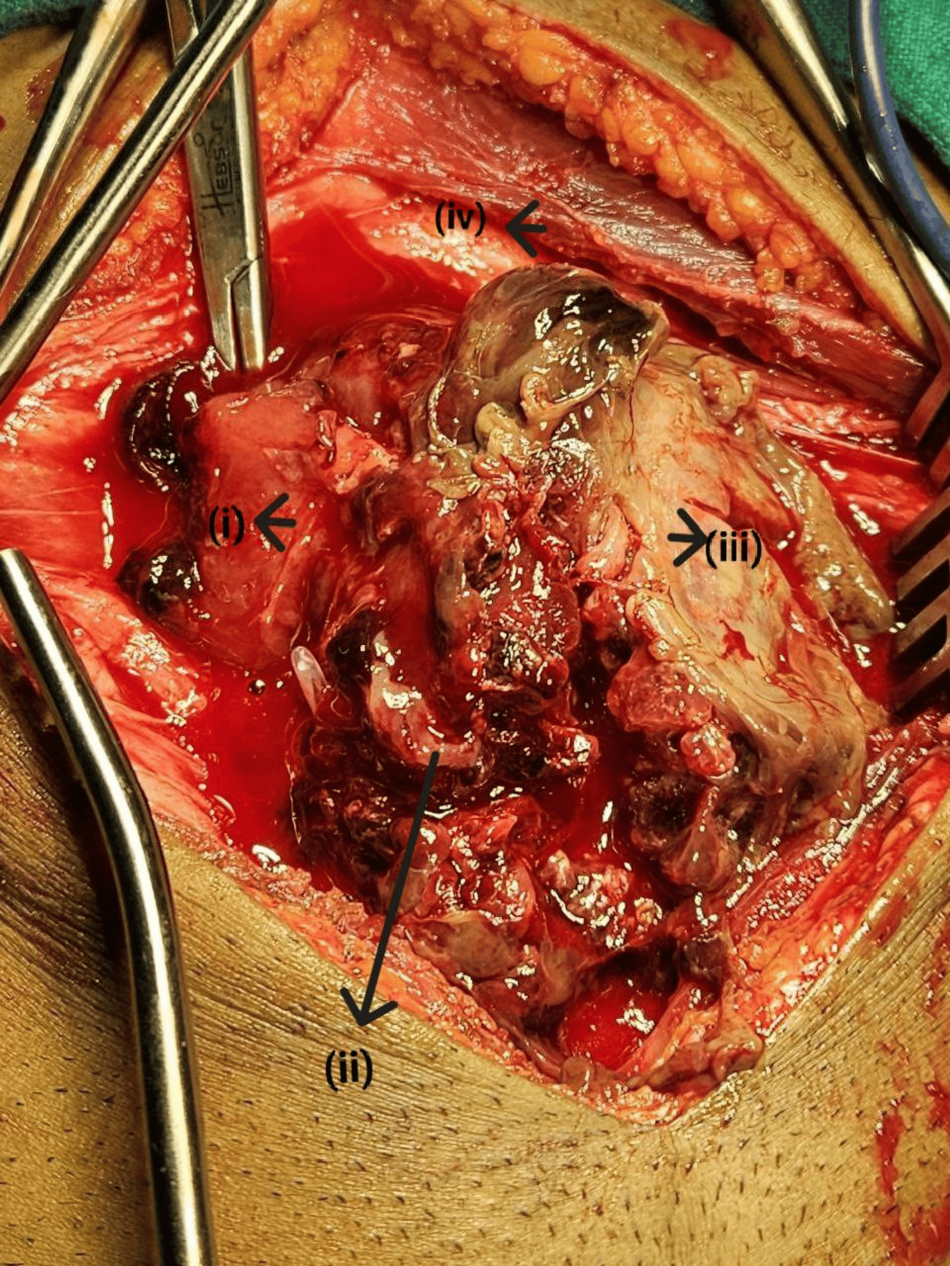
Intraoperative image showing (i) the clot, (ii) hematoma, (iii) bladder tumor, and (iv) rectus sheath

Under general anesthesia, the patient underwent cystoscopy, revealing a trabeculated bladder with multiple clots and a highly ulcerated vascular tumor emerging from the anterolateral wall. Transurethral resection of the bladder tumor was performed, removing approximately 60% of the tumor mass. Tissue samples were then sent for histopathology, which revealed fragments exhibiting spindle to ovoid cell proliferation loosely arranged in a myxoid stroma, indicative of a spindle cell neoplasm consistent with an IMT. After a thorough literature review aimed at the patient's optimal outcome, it was decided to proceed with bladder-conserving surgery. Given the tumor's low metastatic potential but high likelihood of recurrence, a bladder-conserving approach was chosen to ensure complete resection. Under general anesthesia, following scopy, a cystostomy was performed at the dome, targeting the left side (Figure 2).

**Figure 3:**
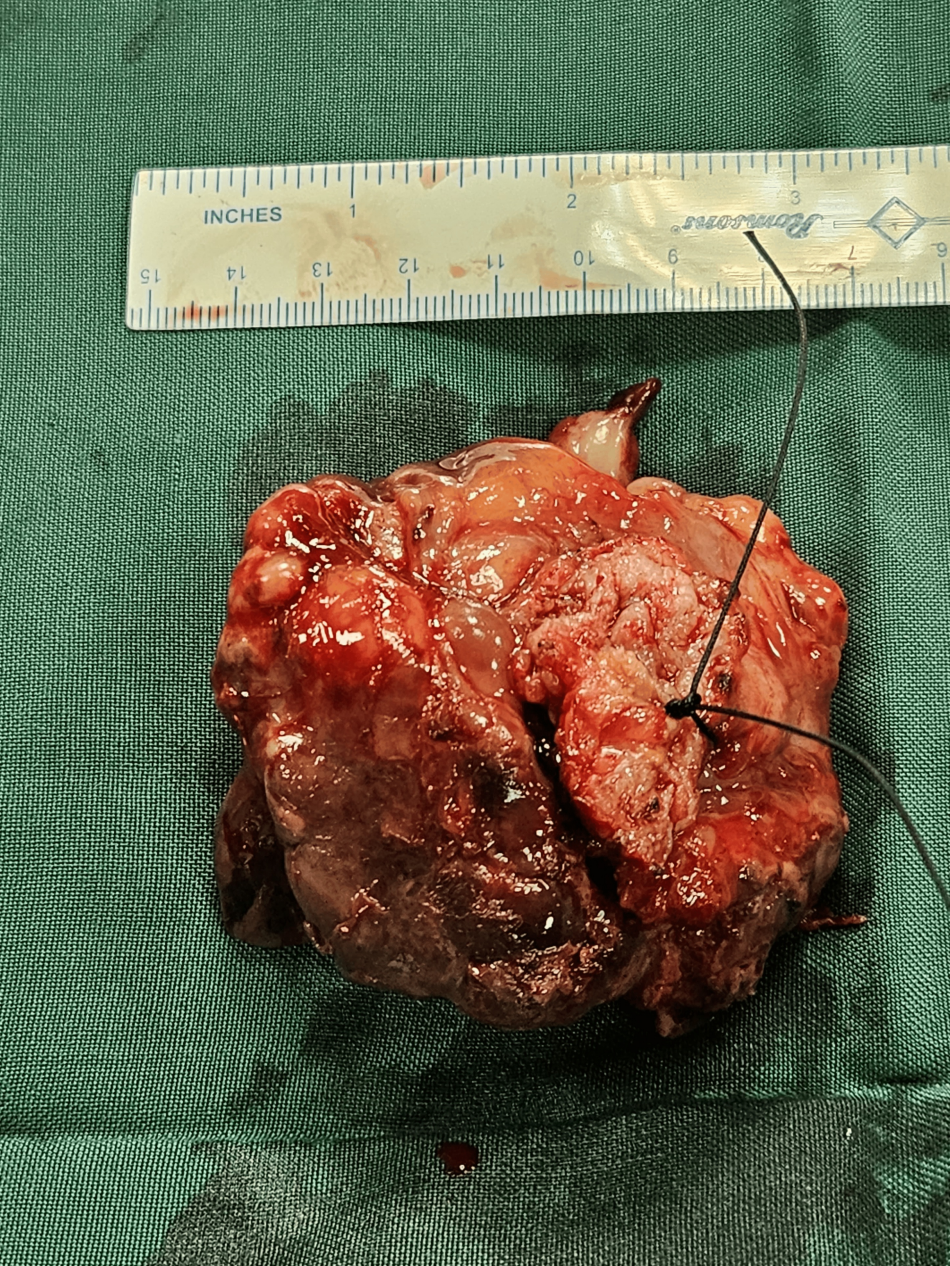
Post Partial Cystectomy Specimen

**Figure 4:**
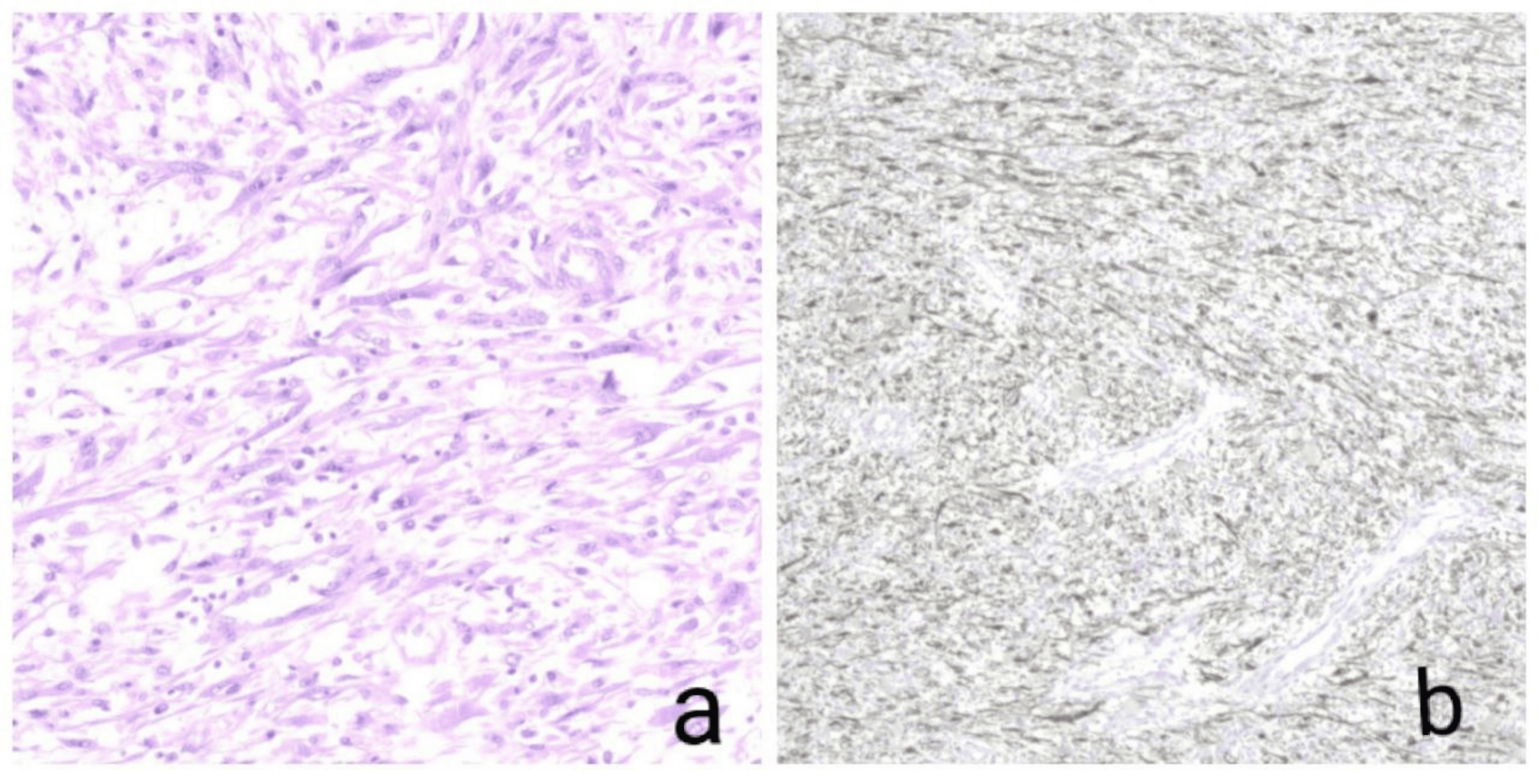
a) Histopathology of the specimen showing spindle shaped myofibroblasts proliferation with no signs of necrosis b) Immunohistochemistry image showing strong positivity for ALK-1.

A complete tumor excision was achieved (Figure 3), followed by closure in layers. Histopathology of the specimen revealed an infiltrating lesion characterized by spindle cell proliferation (Figure 4), with samples from the base indicating involvement of the muscularis propria. Immunohistochemical analysis conducted on the specimen exhibited strong positivity for ALK-1, SMA, and PAN CK and patchy positivity for desmin, indicative of an IMT. The patient underwent rigorous post-operative monitoring, and at the six-month and one-year follow-up post-surgery, there were no discernible indications of recurrence or metastasis.

## Discussion

The IMT, formerly known as the inflammatory pseudotumor, is now delineated as a distinct entity. IMTs are classified as malignant, in contrast to inflammatory pseudotumors, which exhibit a more reactive nature and lack malignant propensity [4]. Bladder IMT remains an exceedingly rare diagnosis, and the literature regarding the optimal diagnosis, management, and follow-up protocol remains scarce [4]. The predominant clinical manifestation of bladder IMTs typically presents as painless gross hematuria, with additional symptoms encompassing obstructive and/or irritative voiding symptoms, abdominal discomfort, and, infrequently, constitutional symptoms such as fever and weight loss.

Immunohistochemically, IMTs demonstrate expression of ALK-1 in 87.5% of cases, SMA in 90% of cases, pancytokeratin focally in over 50% of cases, and desmin in approximately 50% of cases [5]. In this instance, with the specimen testing positive for ALK-1, a favorable prognosis with a low recurrence rate was anticipated based on existing studies. Notably, the largest systematic review on this subject encompassed 182 patients but included both IMTs and pseudosarcomatous myofibroblastic proliferations of the bladder [4].

Management options consisted of transurethral resection of bladder tumor (TURBT) alone, TURBT followed by partial cystectomy and radical cystectomy. TURBT is the initial treatment in over 50% of cases, with subsequent cystectomy, primarily partial, necessitated in 10-20% of cases, predominantly due to incomplete endoscopic resection or if the tumor invades the muscularis [4]. The decision to perform a radical cystectomy rather than a partial cystectomy was mainly made due to the misdiagnosis of the tumor initially and tumor recurrence [4]. In our patient, TURBT removed only 60% of the tumor, which upon histopathology confirmed the diagnosis of IMT, thus necessitating a subsequent partial cystectomy, which further revealed involvement of muscularis propria. Surgeons used an open approach in 74% of the cases when cystectomy was performed [4], similar to our case. On average, the mean follow-up duration was 14 months [4], and our patient has been under follow-up for 12 months now, with no signs of recurrence or metastasis. A total recurrence of 9% and metastasis rate of 4% were recorded [4].

## Conclusions

Given their rarity and diagnostic complexity, bladder IMTs necessitate a comprehensive approach integrating clinical, histopathological, radiological, and biochemical assessments, as no single method suffices to confirm diagnoses. Accurate identification and differentiation of malignant tumors are imperative to prevent misdiagnosis and overtreatment. Laparoscopic resection may be considered if the tumor's size and location permit. Systemic chemotherapy is done in a few cases to shrink the size of the tumor before surgery or due to an initial misdiagnosis. However, the preferred treatment modality remains TURBT, followed by partial cystectomy if necessary. Vigilant follow-up is crucial due to the heightened risk of recurrence in IMT cases. Therefore, management of bladder IMTs encompasses both surgical resection and meticulous surveillance for recurrence.
